# Trajectories of metabolic risk factors and biochemical markers prior to the onset of type 2 diabetes: the population-based longitudinal Doetinchem study

**DOI:** 10.1038/nutd.2017.23

**Published:** 2017-05-08

**Authors:** G Hulsegge, A M W Spijkerman, Y T van der Schouw, S J L Bakker, R T Gansevoort, H A Smit, W M M Verschuren

**Affiliations:** 1 Centre for Nutrition, Prevention and Health Services, National Institute of Public Health and the Environment, Bilthoven, The Netherlands; 2 Julius Center for Health Sciences and Primary Care, University Medical Center Utrecht, Utrecht, The Netherlands; 3 Department of Internal Medicine, Division of Nephrology, University Medical Center Groningen and University of Groningen, Groningen, The Netherlands

## Abstract

**Background:**

Risk factors often develop at young age and are maintained over time, but it is not fully understood how risk factors develop over time preceding type 2 diabetes. We examined how levels and trajectories of metabolic risk factors and biochemical markers prior to diagnosis differ between persons with and without type 2 diabetes over 15–20 years.

**Methods:**

A total of 355 incident type 2 diabetes cases (285 self-reported, 70 with random glucose ⩾11.1 mmol l^−1^) and 2130 controls were identified in a prospective cohort between 1987–2012. Risk factors were measured at 5-year intervals. Trajectories preceding case ascertainment were analysed using generalised estimating equations.

**Results:**

Among participants with a 21-year follow-up period, those with type 2 diabetes had higher levels of metabolic risk factors and biochemical markers 15–20 years before case ascertainment. Subsequent trajectories were more unfavourable in participants with type 2 diabetes for body mass index (BMI), HDL cholesterol and glucose (*P*<0.01), and to a lesser extent for waist circumference, diastolic and systolic blood pressure, triglycerides, alanine aminotransferase, gamma glutamyltransferase, C-reactive protein, uric acid and estimated glomerular filtration rate compared with participants without type 2 diabetes. Among persons with type 2 diabetes, BMI increased by 5–8% over 15 years, whereas the increase among persons without type 2 diabetes was 0–2% (*P*<0.01). The observed differences in trajectories of metabolic risk factors and biochemical markers were largely attenuated after inclusion of BMI in the models. Results were similar for men and women.

**Conclusions:**

Participants with diabetes had more unfavourable levels of metabolic risk factors and biochemical markers already 15–20 years before diagnosis and worse subsequent trajectories than others. Our results highlight the need, in particular, for maintenance of a healthy weight from young adulthood onwards for diabetes prevention.

## Introduction

Although it has been well established that adverse levels of risk factors often develop early in life and are maintained over time,^1, 2, 3, 4, 5, 6^ it is not fully understood how they progress to type 2 diabetes (T2D). For example, T2D might be preceded by a gradual accumulation of the adverse effects of risk factors starting at a young age, or by a relatively sudden deterioration in risk factors before disease onset, or by a combination of both. The comparison of long-term trajectories of risk factors between those who do and those who do not develop T2D may help to identify at which time point these trajectories start to deviate before the development of overt disease. Such insight into the timing and the extent of pathophysiological changes before symptoms occur may provide indications for the optimal timing of preventive actions. Trajectories of BMI and waist circumference are of particular importance since these are strong modifiable risk factors of T2D.^7, 8^ Other relevant factors associated with T2D include glucose levels,^9^ β-cell function,^10^ insulin resistance,^10^ blood pressure,^8^ lipids,^8^ liver fat markers,^11, 12^ markers of chronic inflammation^13^ and kidney function.^14^

Several studies have described gradual changes in β-cell function, insulin resistance, fasting glucose and 2-h post-load glucose many years before diagnosis of T2D with steeper unfavourable changes 3–5 years before diagnosis.^15, 16, 17, 18, 19^ Only a few studies, mainly among men, have examined progressive changes of other risk factors, such as BMI, but so far findings have been inconsistent. The Whitehall II study showed that adults who developed T2D had similar trajectories of BMI and C-reactive protein (CRP) but more unfavourable trajectories of systolic blood pressure and high-density lipoprotein (HDL) cholesterol compared with adults without T2D, over a period of ~14 years.^20, 21^ In contrast, a small study of 177 men observed larger changes in BMI, but no differences in blood pressure, HDL cholesterol and liver fat markers in men who developed impaired fasting glucose compared with men who did not, over a 9-year period.^22^ A short-term study (that is, over 1.5 years) observed differences in changes of alanine aminotransferase (ALT) and triglycerides but not in blood pressure, total cholesterol and HDL cholesterol between high-risk men with incident T2D and controls.^17^

A longer follow-up period in a population-based study and inclusion of other metabolic risk factors and biochemical markers is needed for more insight in the physiological changes preceding the onset of T2D. There is also a need to investigate differences between men and women since previous studies reported several sex-related differences in the associations of risk factors such as systolic blood pressure, HDL cholesterol and uric acid with T2D.^23, 24^ Therefore, we examined whether trajectories of metabolic risk factors and biochemical markers among initially healthy men and women differed for those who developed T2D and those who did not over a period of up to 15–20 years.

## Materials and methods

### Population

The Doetinchem Cohort Study is a population-based longitudinal study of men and women aged 20–59 from Doetinchem, a town in the Netherlands. Men and women were invited for a clinical examination in 1987–1991 (wave 1, *N*=7 768, participation rate: 62%), 1993–1997 (wave two, *N*=6 117), 1998–2002 (wave 3, *N*=4 918), 2003–2007 (wave 4, *N*=4 520) and 2008–2012 (wave 5, *N*=4018). Response rates were 75% or higher in waves 2–5. Details of the study are described elsewhere.^25^ Exclusion criteria were participation in only one wave (*N*=1 378); prevalent diabetes at baseline (*N*=48); missing diabetes status in all waves (*N*=3) and the missing data on biochemical markers in all waves due to absence of informed consent to use blood samples for future research (*N*=122). This led to a population of 2913 men and 3304 women. Pregnant women were excluded for the wave in which they were pregnant. All participants gave written informed consent in each wave and the study was approved by the Medical Ethics Committee of the University Medical Center Utrecht.

### Measurements

Weight, height, waist circumference, diastolic and systolic blood pressure measurements, and blood samples were taken according to standard protocols.^25^ Total cholesterol and HDL cholesterol were measured until 1998 in non-fasting EDTA-plasma and from 1998 onwards in serum, using standardised enzymatic methods. In 2013–2014, standardised enzymatic methods were used to retrospectively determine triglycerides, ALT, gamma glutamyltransferase (GGT), high sensitivity CRP, uric acid, cystatin C and creatinine levels of waves 2–5 for the whole population using blood plasma that had been stored in freezers. Details of all measurements are described in the Supplementary Material. Estimated glomerular filtration rate (eGFR) was calculated using the combination of cystatin C and creatinine.^26^ The data on educational attainment, smoking status and use of anti-hypertensive and cholesterol-lowering medications were obtained by questionnaire.

### Type 2 diabetes

T2D was ascertained by self-report. Of the self-reported cases up to 31 December 2007, 80% were checked with the general practitioner or pharmacist registries (*N*=201):^27^ 176 of these 201 self-reported cases were confirmed as having T2D. All self-reported incident diabetes cases that were not checked (*N*=109) were considered to have type 2. In addition, 70 participants were ascertained as having incident T2D by a measurement of random glucose of ⩾11.1 mmol/l in the physical examination for our study. This gives a total of 355 participants with incident T2D.

### Selection of controls

For each incident T2D case (*N*=355), six controls were randomly selected from the same study wave and matched on age (±2 years) and sex using incidence density sampling, the preferred method for a nested case-control design and recently proposed for retrospective, longitudinal analyses.^28, 29^ We performed age, sex and wave matching to limit the possibility that differences in risk factors due to differences in age and length of follow-up would obscure the differences in risk factors between participants with and without T2D.

### Data analysis

The time of case ascertainment was the first examination wave in which participants reported that they had T2D and/or were found to have a random glucose ⩾11.1 mmol l^−1^. The same wave was used for their matched controls. Participants were followed back in time for 6–21 years, depending on the wave in which they were ascertained as being a T2D case or control (Supplementary Figure 1), that is, participants ascertained in waves 2, 3, 4 or 5 could be followed back in time for 6, 11, 16 or 21 years, respectively. BMI, blood pressure, total cholesterol and HDL cholesterol were followed back in time for a maximum of 21 years. Other risk factors were followed back in time for a maximum of 15 years since those factors were not measured in the first examination wave.

Trajectories preceding case ascertainment were constructed by estimating the marginal means based on the parameter estimates at four or five points in time using linear generalised estimating equation models with an autoregressive correlation structure, separately for each risk factor and marker (dependent variable). T2D status was included as an independent variable in the model to obtain trajectories separately for participants with and without T2D. The model also included the following independent variables: linear, quadratic and cubic terms of age, examination wave, and time as a categorical variable (that is, examination wave). The analyses were stratified by sex. Age was centred at 60 years, which was approximately the mean age at wave 5, and examination wave was centred at wave 5 to fit trajectories for a hypothetical population of 60 year olds in 2008–2012 (T_0_). Trajectories of diastolic and systolic blood pressure were also adjusted for anti-hypertensive medications, and trajectories of total cholesterol, HDL cholesterol and triglycerides for cholesterol-lowering medications. We log-transformed triglycerides, ALT, GGT and CRP and reported geometric means since these biochemical markers did not have a normal distribution.

For participants with a self-reported diagnosis, the date of diagnosis was somewhere between the first wave in which they reported that they had T2D (case ascertainment) and the previous wave. Treatment after diagnosis (that took place in between two successive waves of our study) may have changed the trajectories of participants with a self-reported diagnosis, and would be reflected in the trajectory over the last 5 years before case ascertainment. Therefore, the trajectory over the last 5 years was not taken into account when testing differences between those with and without T2D in the total trajectories of metabolic risk factors and biochemical markers. The time from 15/20 years prior to case ascertainment up to 5 years prior to case ascertainment was used to statistically test differences. This was done using an interaction term for the interaction between the independent variables T2D status and time (dummy relating T_−15/−20_ to T_−__5_), assuming a linear pattern over that period. Differences in trajectories of risk factors and biochemical markers between participants with and without T2D during the last 5 years prior to case ascertainment were also tested using an interaction term between T2D status and time (dummy relating T_−5_ to T_0_). A *P*<0.10 was considered statistically significant for interactions. The analyses were also stratified by method of case ascertainment (that is, self-report and random glucose ⩾11.1 mmol l^−1^) to further investigate the potential effects of medical treatment after the diagnosis of T2D among the self-reported cases during the 5 years preceding case ascertainment. This stratification was done for BMI, systolic blood pressure, total cholesterol and glucose in men. We statistically tested these differences in trajectories by method of case ascertainment using interaction terms between time and method of case ascertainment. To investigate whether differences in trajectories between participants with and without T2D could be explained by BMI, trajectories were additionally adjusted for BMI and centred at 25 kg m^−2^ in sensitivity analyses. To investigate potential misclassification of controls with high random glucose levels, we conducted sensitivity analyses in which we excluded controls with random glucose between 7.0–11.1 mmol l^−1^. All analyses were performed using SAS 9.3 software (Cary, North Carolina, USA).

## Results

In total, 194 men and 161 women developed T2D. In participants with and without T2D, blood pressure, total cholesterol, HDL cholesterol and BMI were followed back in time for an average of 14.0 years, while the other risk factors and biochemical markers were followed back for an average of 10.6 years. At case ascertainment (T_0_), the average age was 60.5 (range: 34–80) for men and 61.2 (range: 33–80) for women. Participants with incident T2D were more likely to have a low level of educational attainment and to be on anti-hypertensive and cholesterol-lowering medication (Table 1).

### Levels of metabolic risk factors and biochemical markers 15–20 years prior to diagnosis

**Figure 1:**
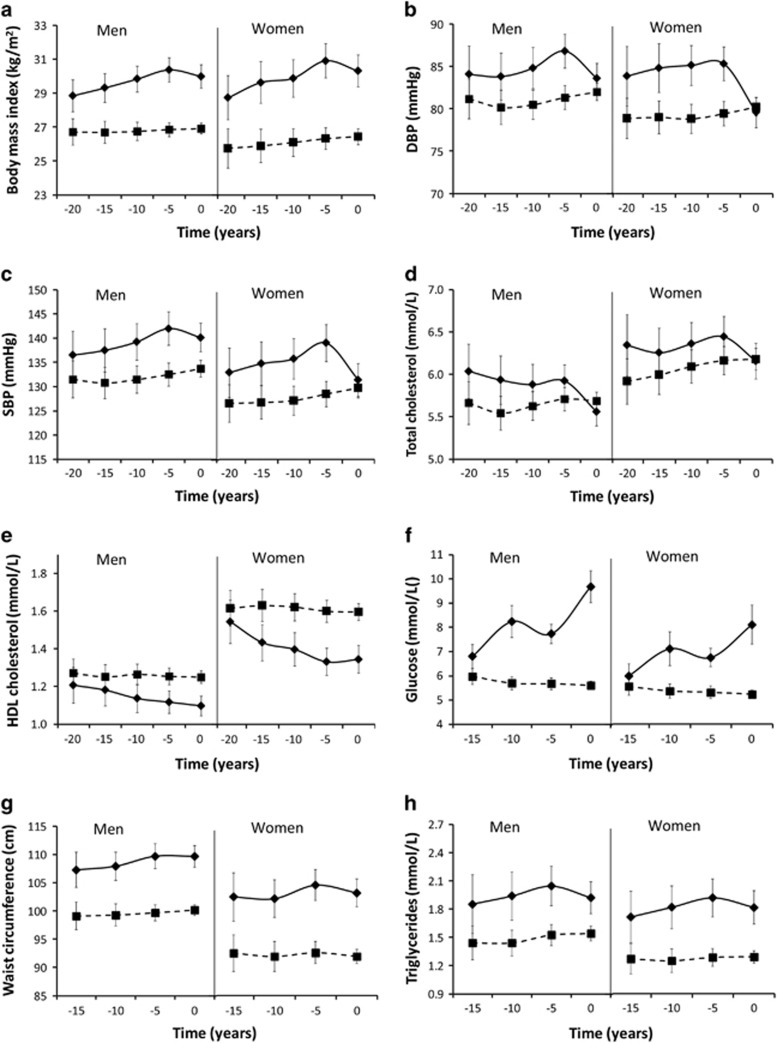

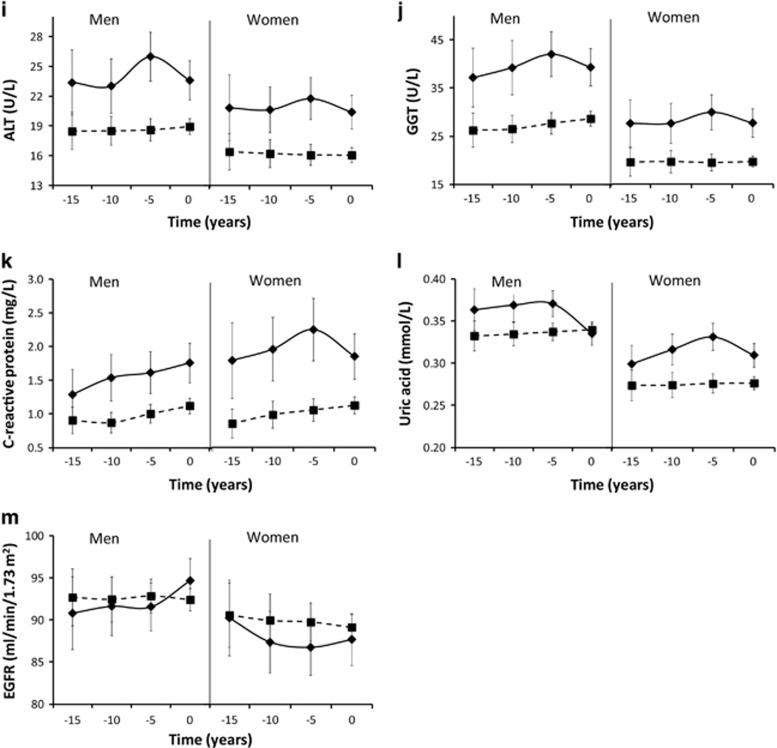
Trajectories of body mass index (**a**), diastolic blood pressure (**b**), systolic blood pressure (**c**), total cholesterol (**d**), HDL cholesterol **(e**), random glucose (**f**), waist circumference (**g**), triglycerides (**h**), alanine aminotransferase (**i**), gamma glutamyltransferase (**j**), C-reactive protein (**k**), Uric acid (**l**) and estimated glomerular filtration rate (**m**) of those with (solid lines) and without (dashed lines) incident type 2 diabetes. Geometric means are shown for triglycerides, alanine aminotransferase, gamma glutamyltransferase and C-reactive protein. ALT, alanine aminotransferase; eGFR, estimated glomerular filtration rate; GGT, gamma glutamyltransferase.

Among those with a 21-year follow-up period, participants with T2D had more unfavourable levels of BMI, diastolic and systolic blood pressure, total cholesterol and HDL cholesterol than those without T2D at 20 years prior to case ascertainment (Figures 1a–e, Supplementary Tables 1–2). Among the same participants, at 15 years prior to case ascertainment, levels of other metabolic risk factors and biochemical markers were similar (and eGFR) or higher (glucose, waist circumference, triglycerides, ALT, GGT, CRP and uric acid) among subjects with T2D than among subjects without T2D (Figures 1f–m, Supplementary Tables 1–2).

### Trajectories

As regards the development in metabolic risk factors and biochemical markers during the 15–20 years prior to case ascertainment, those subjects with incident T2D had in particular larger unfavourable changes over time (that is, unfavourable trajectories) in BMI, HDL cholesterol and random glucose (*P* for interaction<0.01) (Figure 1, Tables 2, Tables 3). For example, BMI increased among men and women with T2D with 1.5 kg m^−2^ (5%) (95% CI: 0.9–2.2) and 2.2 kg m^−2^ (8%) (95% CI: 1.3–3.1), respectively, between T_−__20_ and T_−5_. In contrast, among those without T2D BMI remained stable over time (*P*>0.05). Trajectories of diastolic and systolic blood pressure, waist circumference, triglycerides, ALT, GGT and CRP were also more unfavourable in participants with T2D than in those without T2D, although the difference was not statistically significant for triglycerides, ALT, GGT and CRP. For example, GGT increased borderline significantly with 0.12 log mg l^−1^ (13%) (95% CI: −0.004 to 0.25) among men with T2D and non-significantly with 0.05 log mg l^−1^ (5%) (95% CI: −0.02 to 0.13) among men without T2D between T_−15_ and T_−5_. During the last 5 years before case ascertainment, levels of metabolic risk factors and biochemical markers remained stable or decreased, except for glucose in both sexes and CRP in men.

Trajectories of uric acid and eGFR (that is, declining eGFR) were more unfavourable for women with incident T2D than for women without T2D up to 5 years prior to case ascertainment (*P* for interaction<0.05), whereas there was no significant difference among men (*P* for interaction⩾0.10). Trajectories of total cholesterol were not significantly different between participants with and without incident T2D (*P* for interaction⩾0.10) (Figure 1, Tables 2 and 3).

### Differences in trajectories between cases ascertained by self-report and by random glucose

**Figure 2:**
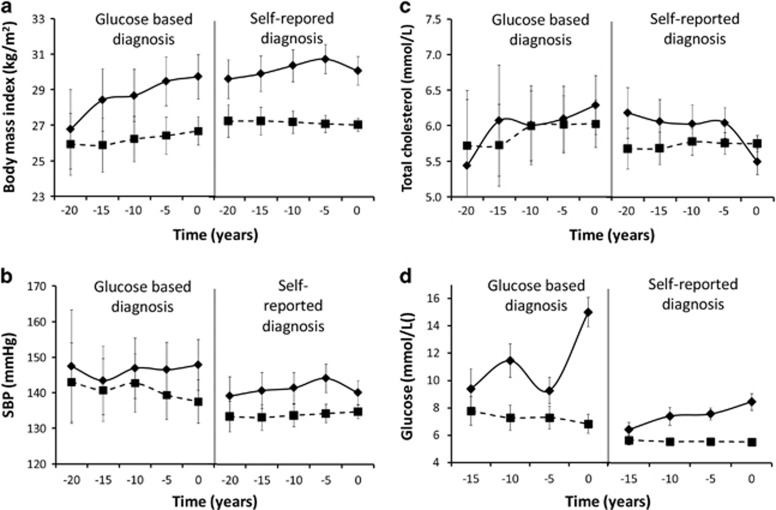
Trajectories of body mass index (**a**), systolic blood pressure (**b**), total cholesterol (**c**) and random glucose (**d**) of those with (solid lines) and without (dashed lines) incident type 2 diabetes, stratified by diagnosis based on glucose ⩾11.1 mmol l^−1^ and self-reported diabetes. Note: time before diagnosis ranged from −17.5 to 2.5 among the self-reported cases since participants were diagnosed somewhere between case ascertainment (year 0) and the previous wave.

Trajectories of BMI, systolic blood pressure, total cholesterol and random glucose were similar for participants ascertained by self-report and those ascertained by elevated random glucose up to 5 years prior to case ascertainment (*P* for interaction ⩾0.10) (Figure 2). In contrast, during the last 5 years prior to case ascertainment, levels of BMI, systolic blood pressure and total cholesterol decreased among participants with self-reported T2D but not among participants diagnosed by elevated random glucose based on our study examination (*P* for interaction<0.10).

### Adjustment for BMI

Adjustment for BMI strongly attenuated differences in trajectories between participants with and without incident T2D for all metabolic risk factors and biochemical markers except for random glucose (Supplementary Figure 2).

### Sensitivity analyses

In sensitivity analyses, exclusion of controls with random glucose levels between 7.0–11.1 mmol l^−1^ resulted in the same findings as our primary results.

## Discussion

Men and women with incident T2D had more unfavourable levels of metabolic risk factors and biochemical markers than those without T2D 15–20 years prior to diagnosis. Subsequent trajectories were also more unfavourable in participants with T2D than in those without T2D for BMI, HDL cholesterol, glucose and to a lesser extent for diastolic and systolic blood pressure, waist circumference, triglycerides, markers of liver fat and chronic inflammation, uric acid and kidney function. The patterns were similar for men and women. Differences in trajectories between participants with and without T2D were much smaller after adjustment for BMI.

A greater decrease in metabolic risk factors and biochemical markers in those with T2D than in controls during the 5 year prior to ascertainment was observed. Since the diagnosis of T2D occurred at an unknown time point during the 5 years preceding case ascertainment, medical treatment and lifestyle changes will have often already started before the wave in which a respondent reported a diagnosis of T2D. This implies that medical treatment and lifestyle intervention after the diagnosis of T2D had a large favourable impact on levels of almost all metabolic risk factors and biochemical markers. This is supported by analyses stratified by method of case ascertainment, which showed that the drop in BMI, systolic blood pressure and total cholesterol was only apparent in men with a self-reported diagnosis (diagnosed in the years before case ascertainment), and not in cases ascertained by elevated random glucose levels during the examination for our study.

Extending earlier findings that unfavourable changes in ALT and triglycerides precede the diagnosis of T2D in men over a 1.5-year period,^17^ we showed that differences in ALT, triglycerides and additionally GGT between men and women with and without incident T2D already exist 10–15 years before the onset of T2D. These differences continue to increase until diagnosis, although no longer at a statistically significantly more unfavourable rate in persons with T2D than in persons without T2D. The increase in ALT and GGT, indicating hepatic fat accumulation, leads to higher concentrations of very low density lipoprotein (VLDL) particles in the circulation, which may lead to hypertriglyceridemia and lower HDL cholesterol.^30^ This is consistent with our observed unfavourable trajectories in triglycerides and HDL cholesterol that occurred concurrently with unfavourable trajectories in ALT and GGT among participants with incident T2D.

The present work also extends previous work on the relation between uric acid and T2D^31^ by showing that unfavourable changes in uric acid precede the diagnosis of T2D in women but not in men over a period of more than 10 years. This is in line with results from a meta-analysis that showed that each ml dl^−1^ increase in uric acid increased the risk of T2D by 28% among women but only by 9% among men.^31^ This indicates that uric acid may be a more important factor for the development of T2D in women than men. The observed sex difference could be related to hormonal differences. For example, uric acid levels in women have been shown to increase due to menopause-related changes in their metabolism^32^ and due to hormone replacement therapy.^33^ Sex differences might also reflect differences in other metabolic risk factors related to uric acid, drug use or dietary patterns. Furthermore, although it is still uncertain whether elevated uric acid is causally related to T2D,^34^ possible mechanisms include increased oxidative stress, low-grade inflammation and endothelial dysfunction, which are all related to the development of T2D.^35, 36, 37^

In line with our findings, the data from the Whitehall II study and the Framingham Heart Study showed that subjects with T2D had higher mean BMI levels than subjects without T2D at 18 and 20 years prior to diagnosis, respectively.^20, 38^ However, we also observed more unfavourable trajectories of BMI among those participants who developed T2D, while the Whitehall II study found no difference in the trajectories of BMI.^20^ Our findings indicate that, independent of whether the subject is overweight, gaining weight is important in the development of T2D. Contrasting findings might be the result of matching on age, sex and examination wave in our study, leading to a stricter adjustment for age and a smaller difference in selective dropout between those participants with and without T2D. This may have led to trajectories of controls that were more favourable in our study than in the Whitehall II study, and thereby to larger differences in trajectories between those subjects with and without T2D.

In general, the incidence of T2D is relatively low before the age of 45 and increases exponentially thereafter, with ~90% of the incident T2D cases being diagnosed after the age of 45.^39, 40, 41^ Since the present study showed that the differences in trajectories of metabolic risk factors and biochemical markers between those with and without incident T2D start to develop more than 15–20 years before diagnosis, this indicates that measures to prevent T2D are already warranted before the age of 25 and onwards. Our results showed particularly unfavourable changes in adiposity, HDL cholesterol and random glucose, and to a lesser extent in blood pressure, triglycerides, markers of liver fat accumulation and chronic inflammation, uric acid and kidney function. Obesity is a major risk factor for dyslipidaemia, hypertension, liver fat accumulation, chronic inflammation and kidney dysfunction,^42, 43, 44, 45^ and BMI largely explained unfavourable trajectories in those metabolic risk factors and biochemical markers among participants with T2D. Thus, our findings highlight the need for lifestyle interventions to promote the maintenance of a healthy weight from young adulthood onwards to reduce the burden of T2D. Our results further suggest that it may be of interest to investigate whether repeated measurements of risk factors can improve risk prediction of T2D.

One of the strengths of the present study is that, we have measured various metabolic risk factors and biochemical markers in a population-based cohort at four or five points in time over a long follow-up period. We were able to describe long-term trajectories for men and women separately. The limitations of the present study include the limited number of participants with T2D with a follow-up period of 15 or 20 years, leading to relatively large 95% confidence intervals for 15 and 20 years prior to case ascertainment. We identified participants with T2D based on self-report or random glucose levels in blood plasma. Most of these self-reported cases were confirmed by the general practitioner or pharmacist and a validation study indicated a high level of accuracy of self-reported diagnosis in our population.^27^ Nevertheless, misclassification may have occurred, including cases not detected by random glucose levels. As our sensitivity analyses showed that exclusion of controls with high random glucose levels had no effect on the results, bias is likely to be limited, and may only have led to a small underestimation if any of the differences in trajectories between participants with and without incident T2D. Furthermore, individuals who participate in cohort studies are generally healthier and better educated than non-responders, and participants who were excluded and those who dropped out during follow-up also had slightly less favourable levels of the investigated risk factors at baseline. This has most likely led to underestimation of the number of participants with T2D and differences in trajectories between people with and without T2D.

Our results showed that metabolic risk factors and biochemical markers were more unfavourable in people with T2D than in people without T2D 15–20 years or more before diagnosis of T2D and that BMI, HDL cholesterol, random glucose and to a lesser extent diastolic and systolic blood pressure, waist circumference, triglycerides, liver fat and inflammatory markers, uric acid and kidney function gradually deteriorate further up to diagnosis. Unfavourable changes in these metabolic risk factors and biochemical markers occurred at the same time and showed a similar pattern in men and women. Differences in trajectories between subjects with and without incident T2D were explained largely by unfavourable changes in BMI among participants with T2D, stressing the importance of maintaining a healthy weight. These findings underscore the need for primary prevention that starts more than 15 years before the diagnosis of T2D, i.e. from young adulthood onwards.

## Figures and Tables

**Table 1:** Population characteristics of those participants with and without incident type 2 diabetes at case ascertainment (T_0_) (1998–2012), stratified by sex

	*Men*		*Women*	
	*T2D* N=*194*	*No T2D* N=*1 164*	*T2D* N=*161*	*No T2D* N=*966*
Age (years), mean (s.d.)	60.4 (8.8)	60.5 (8.9)	61.2 (8.6)	61.2 (8.7)
				
*Educational attainment*				
Low (%)^a^	104 (54%)	531 (46%)	119 (74%)	630 (65%)

*Smoking status*				
Current smoker (%)	38 (20%)	217 (19%)	61 (39%)	183 (19%)
Ex-smoker (%)	118 (61%)	651 (56%)	35 (22%)	369 (38%)
				
*Medication*				
Anti-hypertensive medication (%)	78 (40%)	201 (17%)	86 (54%)	212 (22%)
Cholesterol-lowering medication (%)	68 (35%)	139 (12%)	64 (40%)	98 (10%)
				
*Risk factors and biochemical markers*				
BMI (kg/m^2^), mean (s.d.)	29.6 (4.3)	26.6 (3.2)	30.3 (5.6)	26.5 (4.4)
DBP (mm Hg), mean (s.d.)	84 (11)	83 (10)	80 (9)	81 (10)
SBP (mm Hg), mean (s.d.)	142 (18)	135 (17)	135 (19)	133 (19)
TC (mmol l^−1^), mean (s.d.)	5.2 (1.2)	5.6 (1.0)	5.5 (1.1)	5.9 (1.1)
HDLc (mmol l^−1^), mean (s.d.)	1.09 (0.31)	1.24 (0.33)	1.33 (0.39)	1.56 (0.39)
Random glucose (mmol l^−1^), mean (s.d.)	9.5 (3.7)	5.4 (1.1)	8.0 (4.3)	5.2 (0.9)
WC (cm), mean (s.d.)	109 (11)	100 (9)	103 (13)	92 (11)
TG (mmol l^−1^), median (IQR)	1.9 (1.3–2.7)	1.5 (1.1–2.1)	1.8 (1.4–2.4)	1.3 (1.0–1.7)
ALT (U l^−1^), median (IQR)	22 (17–32)	18 (14–23)	19 (15–23)	15 (12–19)
GGT (U l^−1^), median (IQR)	36 (25–60)	26 (20–39)	25 (17–37)	17 (13–25)
CRP (mg l^−1^), median (IQR)	2.1 (1.1–3.9)	1.2 (0.6–2.5)	2.2 (1.1–4.6)	1.3 (0.6–2.5)
UA (mmol l^−1^), mean (s.d.)	0.34 (0.08)	0.34 (0.07)	0.30 (0.07)	0.27 (0.07)
eGFR (ml min^−1^/1.73 m^2^), mean (s.d.)	92 (18)	89 (16)	87 (20)	87 (15)

Abbreviations: ALT, alanine aminotransferase; BMI, body mass index; CRP, C-reactive protein; DBP, diastolic blood pressure; eGFR, estimated glomerular filtration rate; GGT, gamma glutamyltransferase; HDLc, high-density lipoprotein cholesterol; IQR, interquartile range; SBP, systolic blood pressure; T2D, type 2 diabetes; TC, total cholesterol; TG, triglycerides; UA, uric acid; WC, waist circumference.

a Intermediate secondary education or less.

**Table 2:** Mean change in metabolic risk factors and biochemical markers over time for men with and without incident type 2 diabetes

	*T*_*−15/20*_ *to T*_*−5*_^a^						*T*_*−5*_ *to T*_*0*_					
	*T2D*			*No T2D*			*T2D*			*No T2D*		
	*Beta*	*95% CI*		*Beta*	*95% CI*		*Beta*	*95% CI*		*Beta*	*95% CI*	
BMI (kg m^−2^)	1.5	0.9	2.2	0.1	−0.3	0.6***	−0.4	−0.7	−0.03	0.1	−0.1	0.2***
DBP (mm Hg)	2.8	−0.2	5.7	−0.2	−1.8	1.4*	−3.2	−5.0	0.9	0.6	−0.1	1.4***
SBP (mm Hg)	5.5	0.9	10.1	1.0	−1.4	3.4*	−1.8	−4.7	1.0	1.2	−0.01	2.4**
TC (mmol l^−1^)	−0.1	−0.3	0.1	0.05	−0.1	0.2	−0.4	−0.5	−0.2	0.02	−0.04	0.1***
HDLc (mmol l^−1^)	−0.09	−0.15	−0.03	−0.02	−0.06	0.02**	−0.02	−0.06	0.02	−0.01	−0.02	0.02
Random glucose (mmol l^−1^)	1.5	1.1	1.9	−0.04	−0.1	0.2***	2.3	1.7	2.9	−0.3	−0.1	0.4***
WC (cm)	2.4	0.4	4.4	0.6	−0.7	1.8**	0.03	−1.2	1.3	0.5	−0.2	1.2
TG (LOG mmol l^−1^)	0.10	−0.05	0.24	0.06	−0.02	0.13	−0.06	0.03	−0.15	0.01	−0.03	0.05
ALT (LOG U l^−1^)	0.11	−0.02	0.23	0.01	−0.06	0.07	−0.10	−0.19	−0.01	0.02	−0.02	0.05**
GGT (LOG U l^−1^)	0.12	−0.004	0.25	0.05	−0.02	0.13	−0.07	−0.15	0.02	0.03	−0.01	0.08**
CRP (LOG mg l^−1^)	0.22	−0.03	0.47	0.10	−0.05	0.26	0.09	−0.07	0.24	0.11	0.03	0.19
UA (mmol l^−1^)	0.007	−0.011	0.026	0.005	−0.005	0.015	−0.035	−0.048	−0.023	0.002	−0.003	0.008***
eGFR (ml min^−1^/1.73 m^2^)	0.7	−2.0	3.5	0.2	−1.6	1.9	3.1	1.4	4.8	−0.4	−1.5	0.6***

Abbreviations: ALT, alanine aminotransferase; BMI, body mass index; CRP, C-reactive protein; eGFR, estimated glomerular filtration rate; GGT, gamma glutamyltransferase; DBP, diastolic blood pressure; HDLc, high-density lipoprotein cholesterol; SBP, systolic blood pressure; T2D, type 2 diabetes; TC, total cholesterol; TG, triglycerides; UA, uric acid; WC, waist circumference.

Difference between individuals with incident type 2 diabetes and controls based on the interaction between diabetes status and time: *P<0.10, **P<0.05, ***P<0.01.

a T, time, indicating the number of years before ascertainment of type 2 diabetes or the same point in time for matched controls.

**Table 3:** Mean change in metabolic risk factors and biochemical markers over time for women with and without incident type 2 diabetes

	*T*_*−15/20*_ *to T*_*−5*_^a^						*T*_*−5*_ *to T*_*0*_					
	*T2D*			*No T2D*			*T2D*			*No T2D*		
	*Beta*	*95% CI*		*Beta*	*95% CI*		*Beta*	*95% CI*		*Beta*	*95% CI*	
BMI (kg m^−2^)	2.2	1.3	3.1	0.6	−0.1	1.2***	−0.6	−1.0	−0.2	0.1	−0.2	0.4***
DBP (mm Hg)	1.4	−1.8	4.7	0.5	−1.1	2.2	−5.7	−7.6	−3.9	0.8	−0.04	1.6***
SBP (mm Hg)	6.1	1.5	10.7	−0.9	−4.3	2.4*	−7.6	−11.0	−4.1	1.3	−0.2	2.7***
TC (mmol l^−1^)	0.1	−0.2	0.4	0.3	0.1	0.4	−0.3	−0.4	−0.1	0.01	−0.1	0.1***
HDLc (mmol l^−1^)	−0.21	−0.29	−0.13	−0.02	−0.07	0.03***	0.01	−0.03	0.06	0.003	−0.03	0.02
Random glucose (mmol l^−1^)	1.2	0.8	1.6	0.04	−0.1	0.2***	1.7	0.9	2.4	0.2	0.1	0.4***
WC (cm)	2.1	−0.6	4.7	0.1	−1.6	1.8*	−0.8	−1.9	0.3	−0.6	−1.7	0.4
TG (LOG mmol l^−1^)	0.11	−0.02	0.25	0.01	−0.1	0.1	−0.05	−0.14	0.03	0.005	−0.03	0.05
ALT (LOG U l^−1^)	0.04	−0.10	0.18	−0.02	−0.10	0.06	−0.06	−0.16	0.03	−0.002	−0.0	0.04
GGT (LOG U l^−1^)	0.08	−0.04	0.20	−0.01	−0.10	0.09	−0.08	−0.18	0.03	0.01	−0.04	0.06*
CRP (LOG mg l^−1^)	0.23	−0.06	0.51	0.21	0.03	0.39	−0.19	−0.41	0.02	0.06	−0.04	0.16**
UA (mmol l^−1^)	0.032	0.017	0.047	0.002	−0.007	0.012***	−0.022	−0.033	−0.011	0.0003	−0.006	0.006***
eGFR (ml min^−1^/1.73 m^2^)	−3.5	−6.5	−0.5	−0.8	−2.8	1.2**	0.9	−1.2	3.0	−0.6	−1.9	0.6

Abbreviations: T2D, type 2 diabetes; BMI, body mass index; WC, waist circumference; DBP, diastolic blood pressure; SBP, systolic blood pressure; TC, total cholesterol; HDLc, high-density lipoprotein cholesterol; TG, triglycerides; ALT, alanine aminotransferase; GGT, gamma glutamyltransferase; CRP, C-reactive protein; UA, uric acid; eGFR, estimated glomerular filtration rate.

Differences between individuals with incident type 2 diabetes and controls based on the interaction between diabetes status and time: *P<0.10, **P<0.05, ***P<0.01.

a T: time, indicating the number of years before ascertainment of type 2 diabetes or the same point in time for matched control.
