# Supplementary material for: Trajectories of metabolic risk factors and biochemical markers prior to the onset of type 2 diabetes: the population-based longitudinal Doetinchem study
Source: Nutr Diabetes. 2017 May 8;7(5):e270–. doi: 10.1038/nutd.2017.23 (PMC5518805; doi:10.1038/nutd.2017.23)
Supplement: Supplementary Table 1 [file nutd201723x1.docx]

**Supplemental Table 1.** Difference in metabolic risk factors and biochemical markers for men with and without incident type 2 diabetes at each time point.

|  | T_-20_ |  |  | T_-15_ |  |  | T_-10_ |  |  | | T_-5_ |  |  | | T_0_ | |
| --- | --- | --- | --- | --- | --- | --- | --- | --- | --- | --- | --- | --- | --- | --- | --- | --- |
|  | Beta | 95%CI |  | Beta | 95%CI |  | Beta | 95%CI |  | | Beta | 95%CI |  | | Beta | 95%CI |
| BMI (kg/m^2^), | 2.1 | 1.5, 2.7 | | 2.6 | 2.1, 3.2 | | 3.1 | 2.5, 3.7 | | 3.5 | | 2.9, 4.1 | | 3.1 | | 2.4, 3.7 |
| DBP (mm Hg) | 2.9 | 0.1, 5.7 | | 3.7 | 1.4, 5.9 | | 4.3 | 2.3, 6.4 | | 5.5 | | 3.8, 7.1 | | 1.6 | | -0.02, 3.2 |
| SBP (mm Hg) | 5.0 | 1.1, 8.9 | | 6.8 | 3.3, 10.2 | | 7.8 | 4.9, 10.6 | | 9.5 | | 6.7, 12.3 | | 6.4 | | 3.7, 9.2 |
| TC (mmol/L) | 0.4 | 0.1, 0.6 | | 0.4 | 0.2, 0.6 | | 0.3 | 0.1, 0.4 | | 0.2 | | 0.1, 0.4 | | -0.1 | | -0.3, 0.03 |
| HDLc (mmol/L) | -0.06 | -0.12, -0.01 | | -0.07 | -0.12, -0.02 | | -0.13 | -0.18, -0.07 | | -0.14 | | -0.18, -0.09 | | -0.15 | | -0.20, -0.10 |
| Random glucose (mmol/L) | - | - | | 0.8 | 0.5, 1.2 | | 2.5 | 2.0, 3.1 | | 2.1 | | 1.8, 2.4 | | 4.1 | | 3.5, 4.6 |
| WC (cm) | - | - | | 8 | 6, 10 | | 9 | 7, 10 | | 10 | | 8, 12 | | 10 | | 8, 11 |
| TG (mmol/L) | - | - | | 0.25 | 0.11, 0.40 | | 0.30 | 0.19, 0.41 | | 0.29 | | 0.20, 0.38 | | 0.22 | | 0.13, 0.31 |
| ALT (U/L) | - | - | | 0.24 | 0.11, 0.36 | | 0.22 | 0.11, 0.32 | | 0.33 | | 0.25, 0.42 | | 0.22 | | 0.14, 0.30 |
| GGT (U/L) | - | - | | 0.35 | 0.23, 0.47 | | 0.39 | 0.28, 0.51 | | 0.42 | | 0.32, 0.51 | | 0.32 | | 0.22, 0.42 |
| CRP (mg/L) | - | - | | 0.36 | 0.10, 0.61 | | 0.57 | 0.38, 0.75 | | 0.47 | | 0.30, 0.65 | | 0.45 | | 0.28, 0.62 |
| UA (mmol/L) | - | - | | 0.031 | 0.013, 0.049 | | 0.034 | 0.019, 0.049 | | 0.033 | | 0.021, 0.046 | | -0.005 | | -0.018, 0.009 |
| eGFR (ml/min/1.73 m^2^) | - | - | | -1.9 | -4.6, 0.9 | | -0.8 | -3.2, 1.6 | | 1.3 | | -3.6, 1.0 | | 2.3 | | -0.3, 4.8 |

Abbreviations: T2D, type 2 diabetes; BMI, body mass index; WC, waist circumference; DBP, diastolic blood pressure; SBP, systolic blood pressure; TC, total cholesterol; HDLc, high-density lipoprotein cholesterol; TG, triglycerides; ALT, alanine aminotransferase; GGT, gamma glutamyltransferase; CRP, C-reactive protein; UA, uric acid; eGFR, estimated glomerular filtration rate.
