# Supplementary material for: Trajectories of metabolic risk factors and biochemical markers prior to the onset of type 2 diabetes: the population-based longitudinal Doetinchem study
Source: Nutr Diabetes. 2017 May 8;7(5):e270–. doi: 10.1038/nutd.2017.23 (PMC5518805; doi:10.1038/nutd.2017.23)
Supplement: Supplementary Table 2 [file nutd201723x2.docx]

**Supplemental Table 2.** Difference in metabolic risk factors and biochemical markers for women with and without incident type 2 diabetes at each time point.

|  | T_-20_ |  |  | T_-15_ |  |  | T_-10_ |  |  | | T_-5_ |  |  | T_0_ |  |
| --- | --- | --- | --- | --- | --- | --- | --- | --- | --- | --- | --- | --- | --- | --- | --- |
|  | Beta | 95%CI |  | Beta | 95CI % |  | Beta | 95%CI |  | | Beta | 95%CI | Beta | | 95%CI |
| BMI (kg/m^2^), | 3.0 | 2.2, 3.8 | | 3.7 | 2.9, 4.5 | | 3.8 | 2.9, 4.6 | | 4.6 | | 3.7, 5.5 | 3.9 | | 3.0, 4.8 |
| DBP (mm Hg) | 5.0 | 1.9, 8.0 | | 5.8 | 3.4, 8.2 | | 6.3 | 4.4, 8.2 | | 5.9 | | 4.2, 7.5 | -0.6 | | -2.3, 1.0 |
| SBP (mm Hg) | 6.3 | 2.4, 10.2 | | 8.0 | 4.8, 11.2 | | 8.6 | 5.4, 11.8 | | 10.5 | | 7.5, 13.6 | 1.7 | | -1.4, 4.7 |
| TC (mmol/L) | 0.4 | 0.2 ,0.7 | | 0.3 | 0.1, 0.5 | | 0.3 | 0.1, 0.5 | | 0.3 | | 0.1, 0.5 | -0.02 | | -0.2, 0.2 |
| HDLc (mmol/L) | -0.07 | -0.14, -0.001 | | -0.20 | -0.26, -0.14 | | -0.22 | -0.28, -0.17 | | -0.27 | | -0.32, -0.22 | -0.25 | | -0.32, -0.19 |
| Random glucose (mmol/L) | - | - | | 0.4 | 0.1, 0.7 | | 1.7 | 1.2, 2.3 | | 1.4 | | 1.2, 1.7 | 2.9 | | 2.1, 3.6 |
| WC (cm) | - | - | | 10 | 7, 13 | | 10 | 8, 13 | | 12 | | 10, 14 | 11 | | 9, 14 |
| TG (mmol/L) | - | - | | 0.30 | 0.17, 0.43 | | 0.37 | 0.27, 0.48 | | 0.40 | | 0.31, 0.49 | 0.34 | | 0.24, 0.44 |
| ALT (U/L) | - | - | | 0.24 | 0.11, 0.37 | | 0.24 | 0.15, 0.33 | | 0.30 | | 0.22, 0.39 | 0.24 | | 0.16, 0.32 |
| GGT (U/L) | - | - | | 0.34 | 0.22, 0.46 | | 0.34 | 0.23, 0.45 | | 0.43 | | 0.33, 0.53 | 0.34 | | 0.23, 0.45 |
| CRP (mg/L) | - | - | | 0.74 | 0.46, 1.01 | | 0.68 | 0.49, 0.88 | | 0.75 | | 0.57, 0.94 | 0.50 | | 0.31, 0.69 |
| UA (mmol/L) | - | - | | 0.025 | 0.011, 0.040 | | 0.042 | 0.030, 0.055 | | 0.055 | | 0.042 ,0.069 | 0.033 | | 0.019, 0.047 |
| eGFR (ml/min/1.73 m^2^) | - | - | | -0.3 | -3.1, 2.4 | | -2.6 | -5.0, -0.1 | | -3.0 | | -5.8, -0.2 | -1.4 | | -4.6, 1.7 |

Abbreviations: T2D, type 2 diabetes; BMI, body mass index; WC, waist circumference; DBP, diastolic blood pressure; SBP, systolic blood pressure; TC, total cholesterol; HDLc, high-density lipoprotein cholesterol; TG, triglycerides; ALT, alanine aminotransferase; GGT, gamma glutamyltransferase; CRP, C-reactive protein; UA, uric acid; eGFR, estimated glomerular filtration rate.
