# Supplementary material for: Trajectories of metabolic risk factors and biochemical markers prior to the onset of type 2 diabetes: the population-based longitudinal Doetinchem study
Source: Nutr Diabetes. 2017 May 8;7(5):e270–. doi: 10.1038/nutd.2017.23 (PMC5518805; doi:10.1038/nutd.2017.23)
Supplement: Supplementary Methods [file nutd201723x3.docx]

**Supplementary methods**

At each examination, body weight was measured to the nearest 0.1 kg on calibrated scales and 1 kg was subtracted to adjust for clothing. Height was measured to the nearest 0.5 cm. BMI was calculated as weight divided by height squared (kg/m^2^). Waist circumference was measured twice to the nearest 0.5 cm, at the level midway between the lowest rib and the iliac crest at the end of expiration, with participants in standing position. The mean of two measures was used for analysis. Diastolic and systolic blood pressure levels were measured twice after 2 minutes of rest and the average of these two measurements was used in the analyses. Participants were measured in sitting position with a random zero sphygmomanometer (Hawksley and Sons, Lancing, UK) in waves one to three. In waves four and five a Speidel Keller meter (Welch Allyn, Skaneateles Falls, NY, USA) was used. Mean diastolic and systolic blood pressure levels measured at wave four were unexpectedly higher compared to the blood pressure levels in the previous and following waves. No clear cause could be identified, therefore, we statistically corrected blood pressure values of wave 4, as described extensively elsewhere.^1^ Total cholesterol and HDL cholesterol were measured until 1998 in non-fasting EDTA-plasma and from 1998 onwards in serum at the Lipid Reference Laboratory of the University Hospital Dijkzigt in Rotterdam, using standardised enzymatic methods. In 2013-2014, standardised enzymatic methods (Roche/Hitachi Modular P analyzer, Mannheim, Germany) were used to retrospectively determine biochemical markers from waves 2-5 in non-fasting plasma samples that had been stored at -20 degree Celsius until June 1995 and at -80 degree Celsius from July 1995 onwards. Gamma-glutamyltransferase (GGT), uric acid, triglycerides (GPO-PAP assay) and alanine aminotransferase (ALT) (kinetic UV assay) were measured with a colorimetric method. ALT measurements until June 1995 were recoded as missing (N=2,495) because during those years blood plasma was stored at -20 degree Celsius, a temperature at which ALT has poor stability.^2^ GGT (N≤29) and ALT (N≤2) values greater than three times the upper normal reference were recoded as missing for that wave since this may indicate liver problems.^3^ High sensitivity CRP was measured with the principle of particle-enhanced immunological agglutination (Tina-quant CRP). CRP values above 10 mg/L were recoded as missing for that wave because this may indicate an acute-phase response to infection for example or physical injury rather than chronic subclinical inflammation (N≤80).^4^ Cystatin C measurement was based on a particle enhanced-turbidimetric immunoassay using reagents from Gentian (Gentian, Moss, Norway) and creatinine was measured with a Creatinine Plus assay (IDMS traceable).

**Supplementary references**

1. Hulsegge G, van der Schouw YT, Smit HA, Daviglus ML, Verschuren WMM. Determinants of attaining and maintaining a low cardiovascular risk profile – the Doetinchem Cohort Study. *Eur J Public Health* 2015;**26**(1):135-140.

2. Williams KM, Williams AE, Kline LM, Dodd RY. Stability of serum alanine aminotransferase activity. *Transfusion* 1987;**27**:431-433.

3. Thapa BR and Walia A. Liver function tests and their interpretation. *Indian J Pediatr* 2007;**74**(7): 663-71.

4. Yeh ET and Willerson JT. Coming of age of C-reactive protein: using inflammation markers in cardiology. *Circulation* 2003;**107**(3):370-1.
