# Supplementary material for: Trajectories of metabolic risk factors and biochemical markers prior to the onset of type 2 diabetes: the population-based longitudinal Doetinchem study
Source: Nutr Diabetes. 2017 May 8;7(5):e270–. doi: 10.1038/nutd.2017.23 (PMC5518805; doi:10.1038/nutd.2017.23)
Supplement: Supplementary Figure 1 [file nutd201723x4.docx]

**
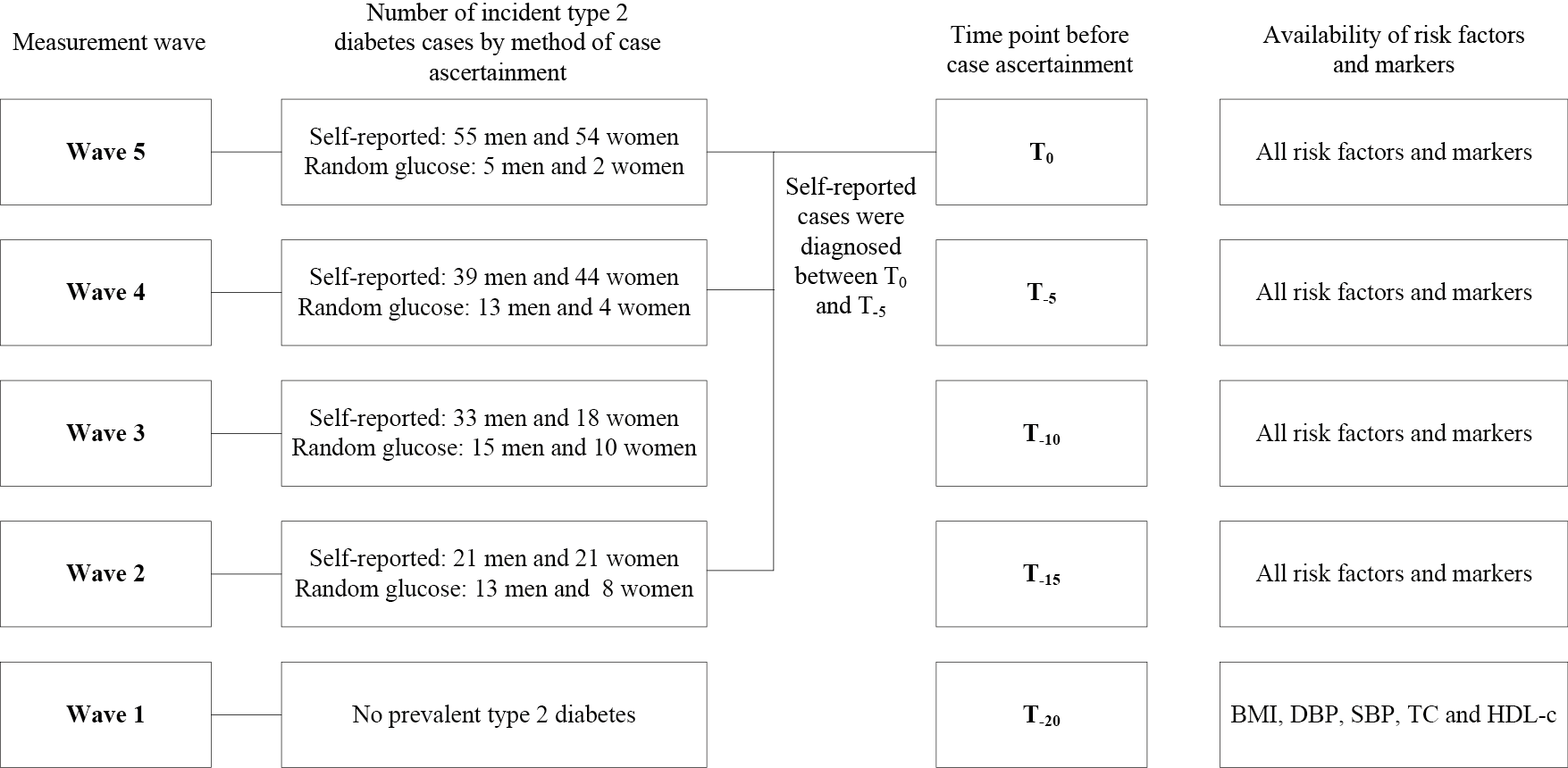
**

**Supplementary Figure 1**. Flow chart of incident type 2 diabetes cases at each wave leading to the study population at case ascertainment (T_0_).

Abbreviations: BMI, body mass index; DBP, diastolic blood pressure; SBP, systolic blood pressure; TC, total cholesterol; HDLc, high-density lipoprotein cholesterol.

Note: for example, cases ascertained at wave 5 (T_0_) had measurements of BMI, DBP, SBP, TC and HDLc for up to 20 years and measurements of other risk factors up to 15 years prior to diagnosis. Cases ascertained at wave 3 (T_0_) had measurements of BMI, DBP, SBP, TC and HDLc for up to 10 years and measurements of other risk factors up to 5 years prior to case ascertainment.

**
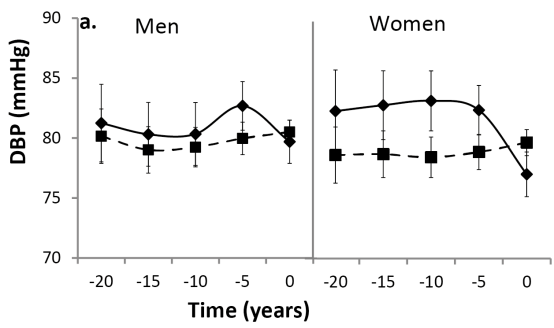

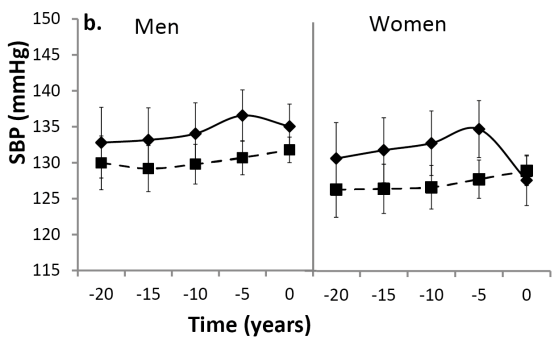

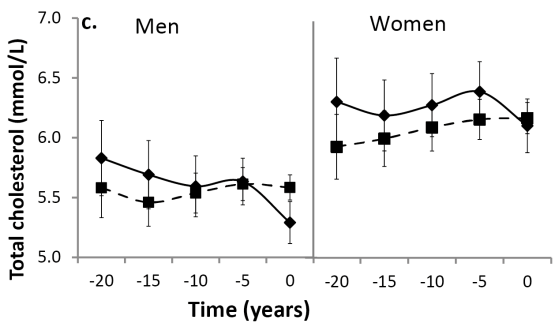

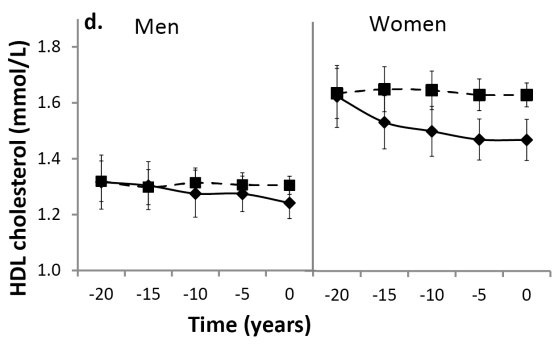
**

**
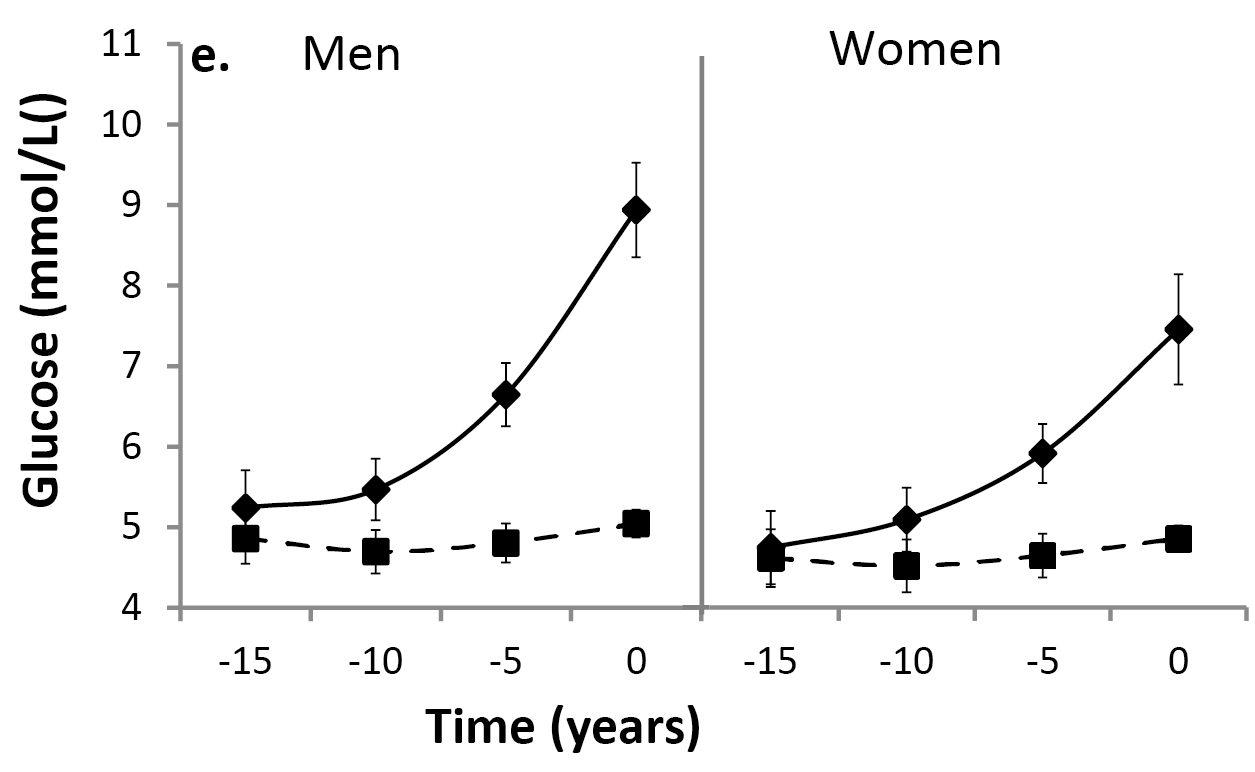

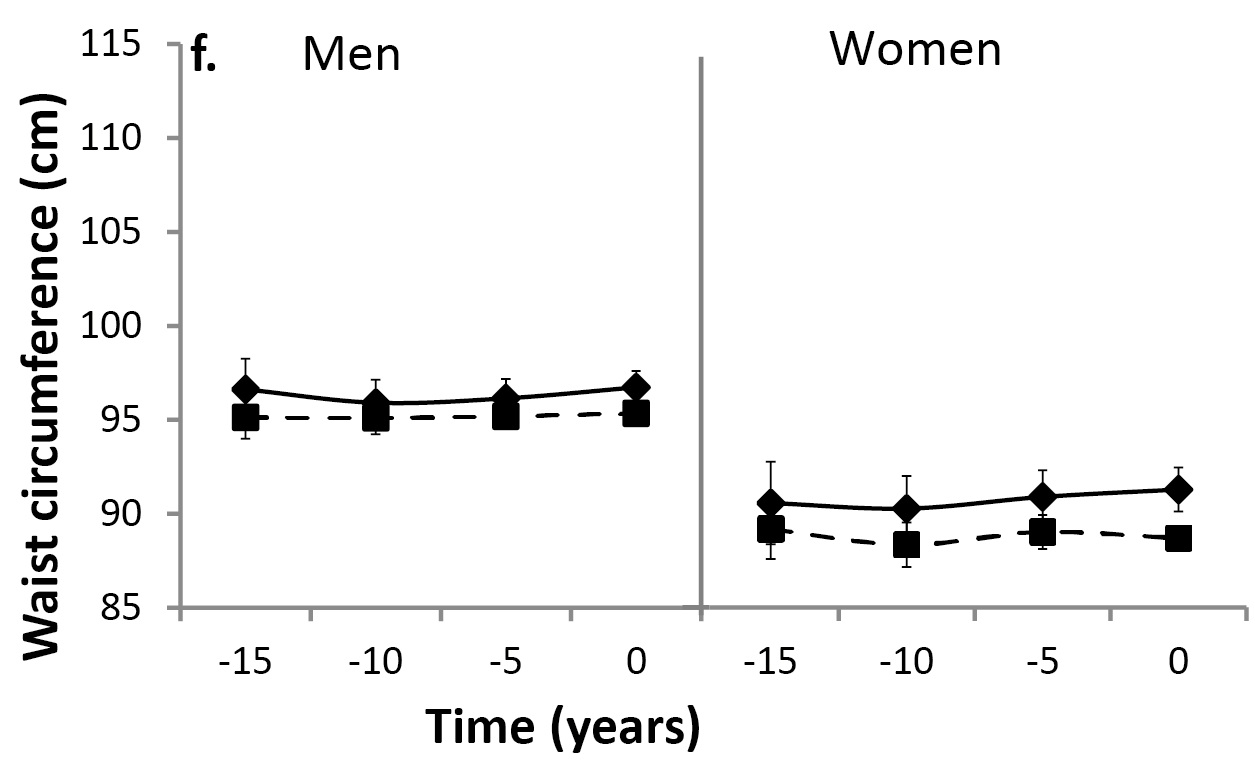

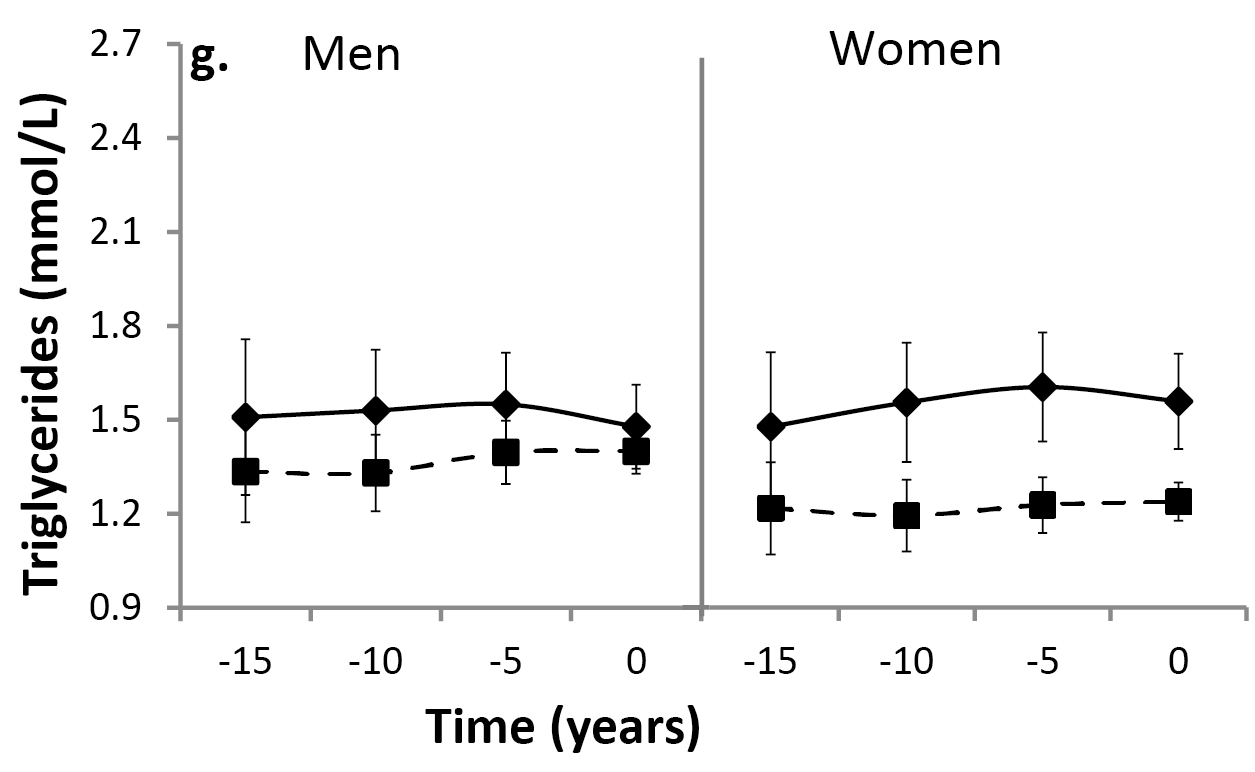

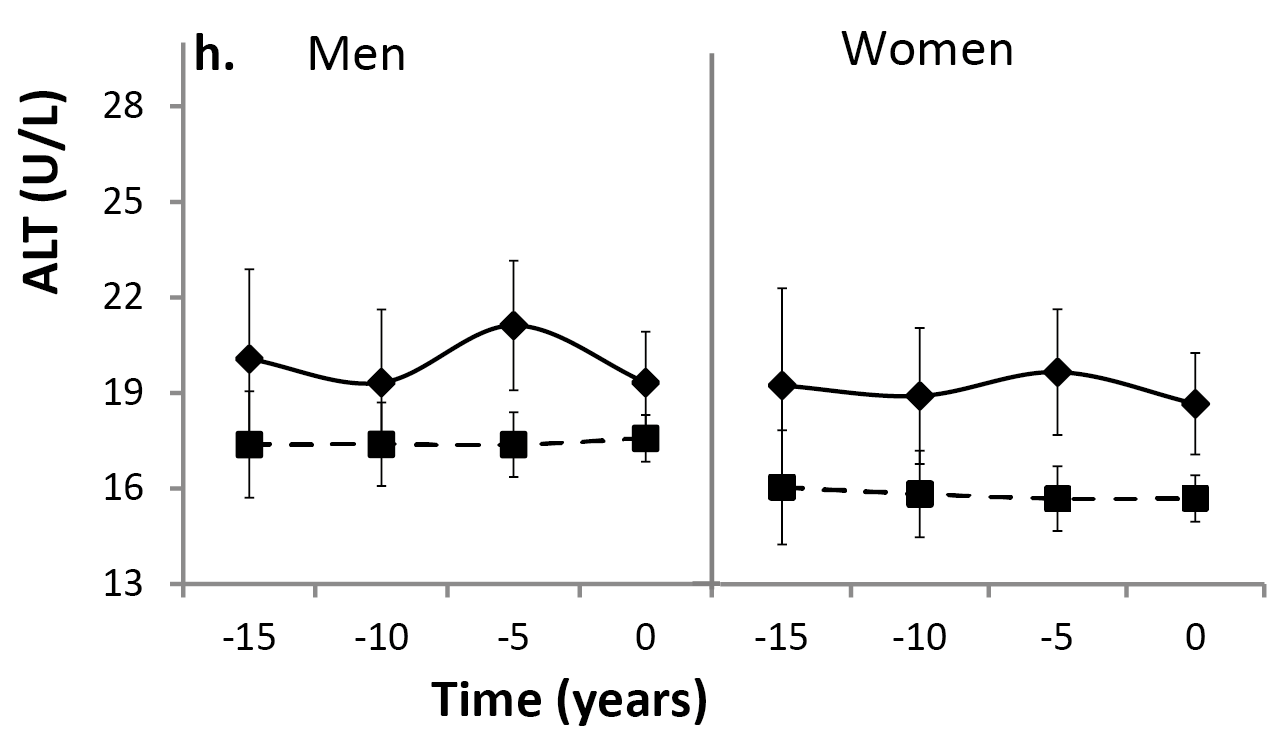
**

**Supplementary Figure 2.** Continues.

**
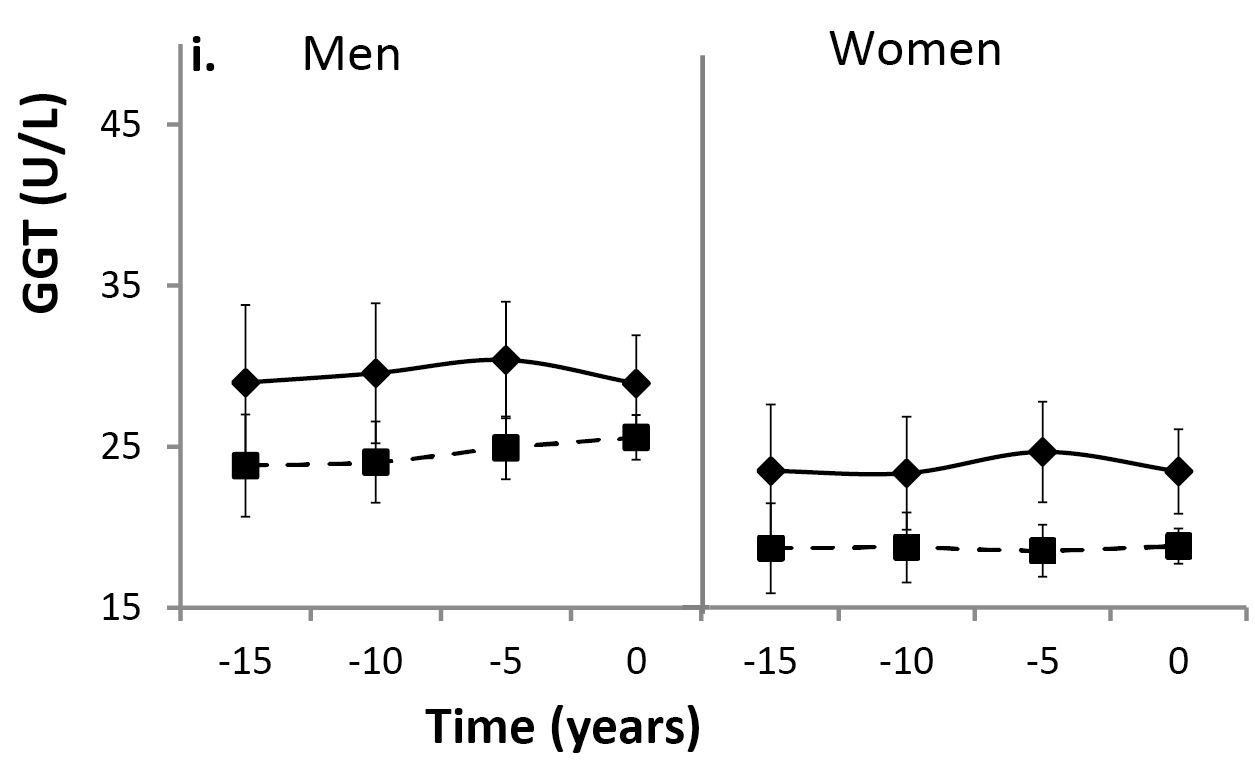

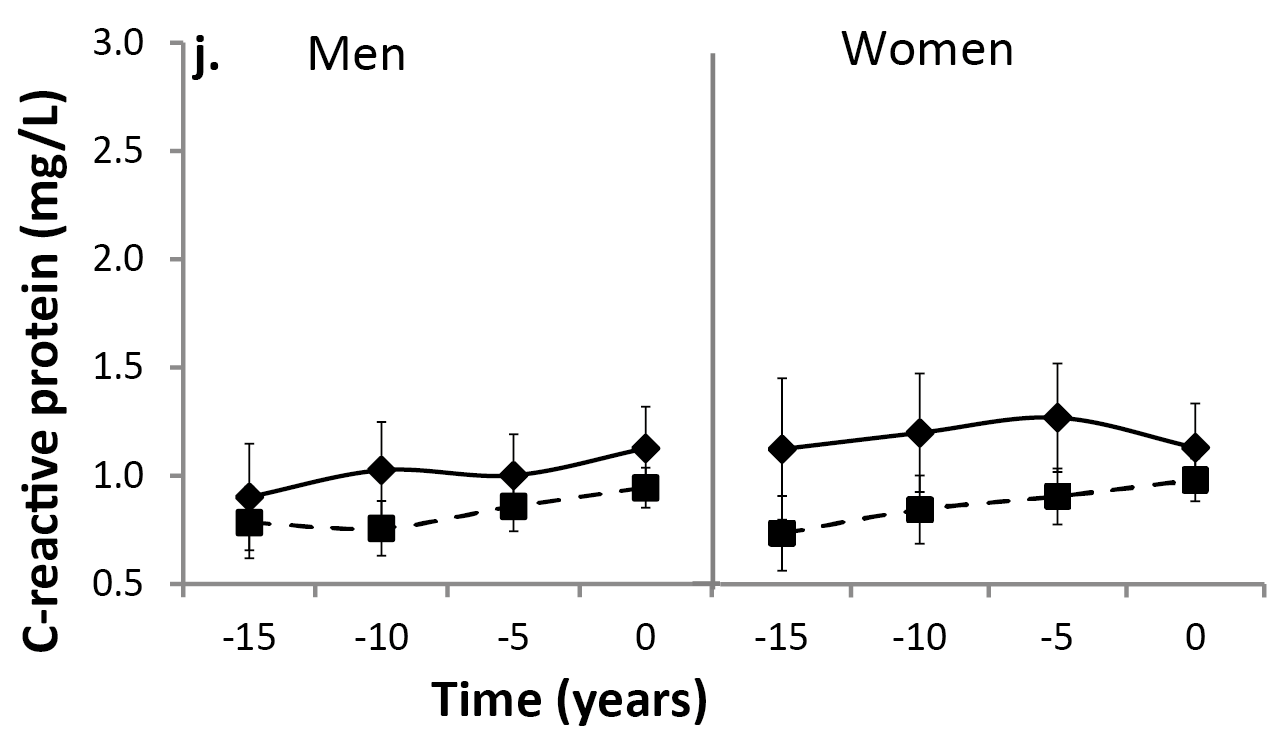

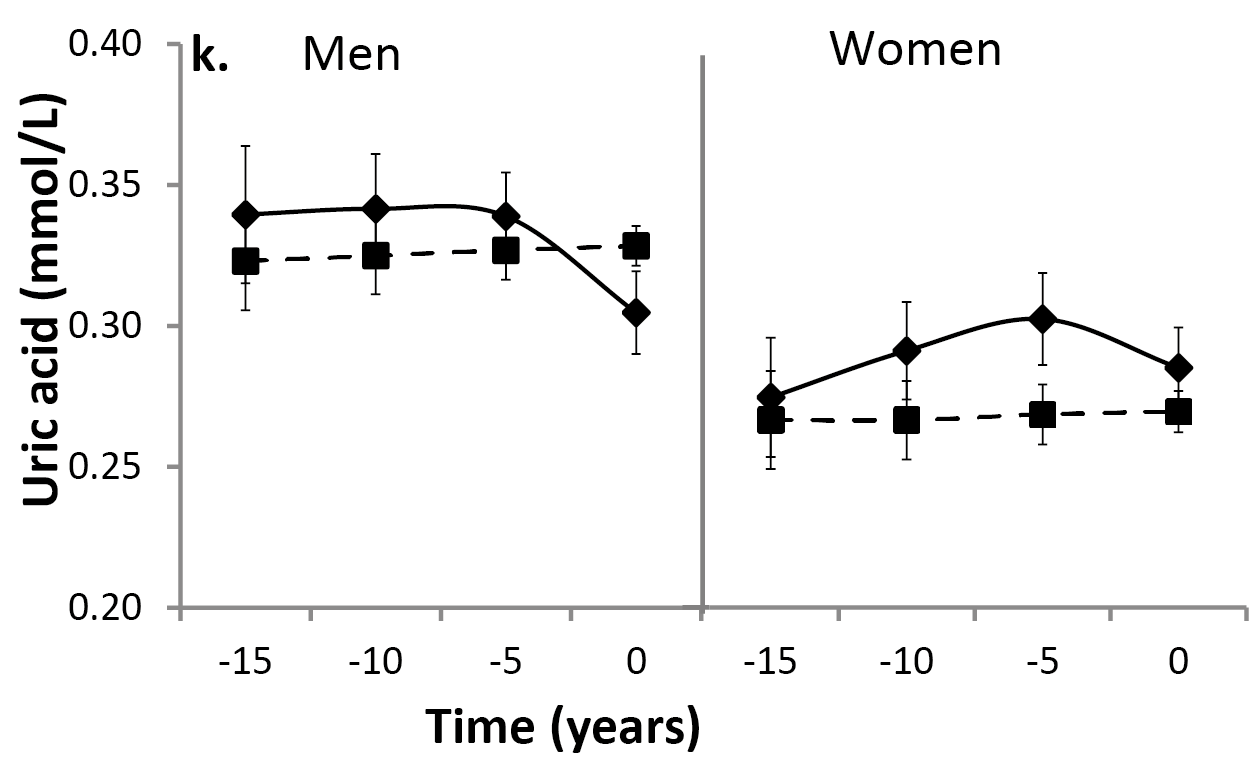

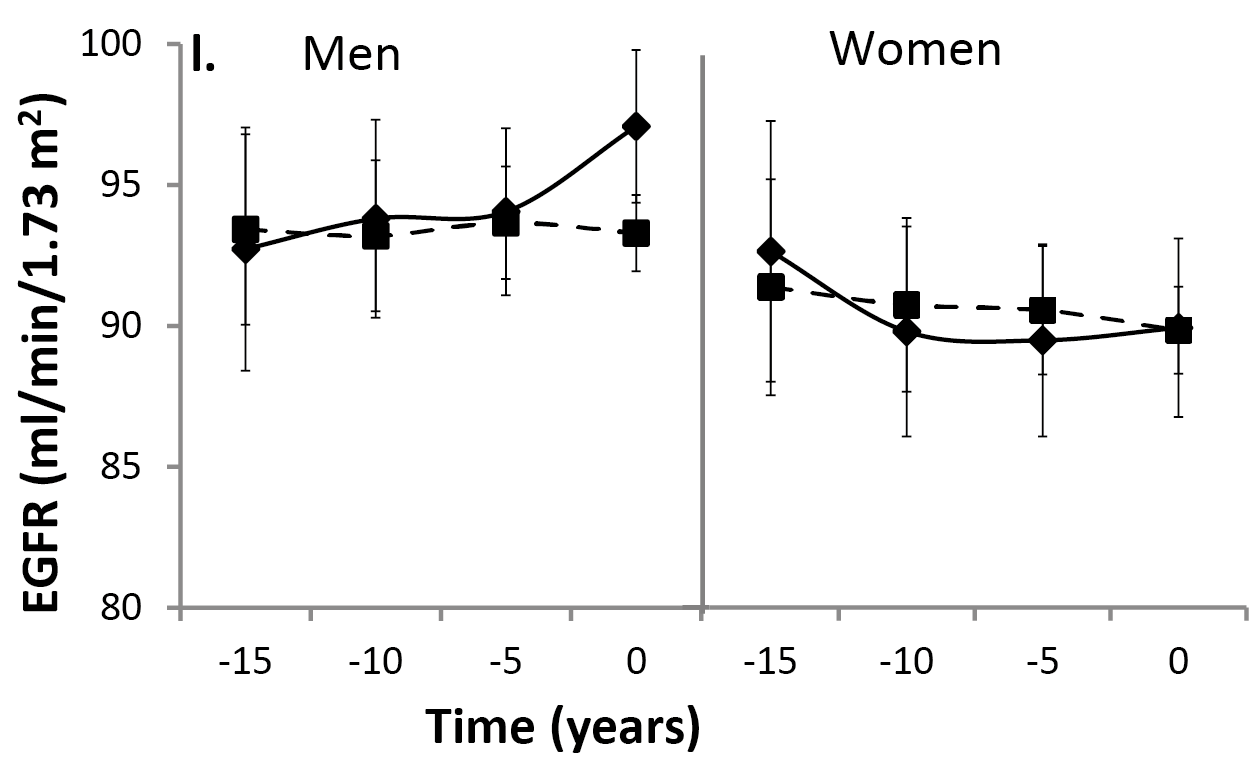
**

**Supplementary Figure 2.** Results adjusted for body mass index. Trajectories of diastolic blood pressure (a), systolic blood pressure (b), total cholesterol (c), HDL cholesterol (d), random glucose (e), waist circumference (f), triglycerides (g), alanine aminotransferase (h), gamma glutamyltransferase (i), C-reactive protein (j), Uric acid (k), and estimated glomerular filtration rate (l) of those with (solid lines) and without (dashed lines) incident type 2 diabetes for men and women who were hypothetically 60 years at the time of case ascertainment.

Abbreviations: DBP, diastolic blood pressure; SBP, systolic blood pressure; ALT, alanine aminotransferase; GGT, gamma glutamyltransferase; eGFR, estimated glomerular filtration rate. Geometric means are shown for triglycerides, alanine aminotransferase, gamma glutamyltransferase and C-reactive protein.
